# Number of Persistent Organic Pollutants Detected at High Concentrations in Blood Samples of the United States Population

**DOI:** 10.1371/journal.pone.0160432

**Published:** 2016-08-10

**Authors:** José Pumarega, Magda Gasull, Duk-Hee Lee, Tomàs López, Miquel Porta

**Affiliations:** 1 Hospital del Mar Medical Research Institute (IMIM), Barcelona, Spain; 2 CIBER de Epidemiología y Salud Pública (CIBERESP), Madrid, Spain; 3 School of Medicine, Universitat Autònoma de Barcelona, Catalonia, Spain; 4 Department of Preventive Medicine, School of Medicine, Kyungpook National University, Daegu, Korea; University of Missouri, UNITED STATES

## Abstract

Human exposure to environmental chemicals as persistent organic pollutants (POPs) is usually assessed considering each pollutant individually, with little attention to concentrations of mixtures in individuals or social groups. Yet, it may be relatively common for humans to have low and high concentrations of numerous POPs. The study objectives were to analyze the number of POPs detected per person at high concentrations in the U.S. population, and the associations between such type of indicators and socioeconomic factors as gender, race / ethnicity, education, and poverty level. From 91 POPs analyzed in serum samples of 4,739 individuals in three subsamples of the National Health and Nutrition Examination Survey (NHANES) 2003–2004 (the last period with valid updated individual data for the compounds considered in the present study), we computed the number of POPs whose serum concentrations were above selected cutoff points. POPs included were 13 organochlorine compounds (OCs), 10 polybrominated diphenyl ethers (PBDEs), the polybrominated biphenyl (PBB) 153, 38 polychlorinated biphenyls (PCBs), 17 polychlorinated dibenzo-*p*-dioxins and dibenzofurans (PCDDs/Fs), and 12 perfluorinated compounds (PFCs). Over 13% of participants had ≥10 of the 37 most detected POPs each at a concentration in the top decile (P90). Over 30% of subjects with total toxic equivalency (TEQ) ≥P75, had ≥10 of 24 POPs not included in TEQ calculations at concentrations ≥P90. Compared to non-Hispanic whites, the adjusted odds ratio of having ≥10 of the 37 POPs at P90 was 9.2 for non-Hispanic blacks and 0.18 for Mexican Americans. Poverty, body mass index, age, and gender were also independently associated with having ≥10 POPs in the top decile. More than one tenth of the US population may have ≥10 POPs each at concentrations in the top decile. Such pattern is nine times more frequent in Non-Hispanic blacks and four times less frequent in Mexican Americans than in non-Hispanic whites.

## Introduction

There is abundant evidence worldwide on lifelong human contamination from mixtures of environmental chemicals as persistent organic pollutants (POPs) [1–7]; yet, the vast majority of studies report each pollutant individually, with little attention to concentrations of mixtures in individual persons or social groups. Thus, the complex features of such internal, body contamination remain unsatisfactorily characterized. Biomonitoring surveys, for instance, do not integrate the number of compounds detected per person and the concentration of each compound [1,4–6,8–12]. Possible health effects of POPs include a variety of developmental, metabolic, neurodegenerative, and neoplastic disorders [5,8,9,10,12–24]. Reasonable concerns exist about such effects at low concentrations; such issues can be integrated with the fact that it is common for humans to have mixtures of POPs at low and high concentrations [6,8,12].

Approaches to these issues include ‘Environment-Wide Association Studies’ (EWAS), and analyses of concentrations of POPs combined, using estimates of total body burden, or different sums of concentrations [24–27]. Efforts to improve exposure assessment must continue: not only to advance etiologic studies and risk assessment, but also to foster knowledge on the characteristics of human chemical contamination itself. Such knowledge is a recognized right of citizens in democratic societies; it is also essential to evaluate the impacts of health, industrial, and related policies [1,5,8,10,28,29]. Indeed, the sources and pathways of exposure to pollutants are socioeconomic and cultural. Thus, strong relationships exist between concentrations of individual POPs and social factors, including income, education, and race / ethnicity [1,30–39]. Unfortunately, such relationships have seldom been analyzed integrating several compounds and their concentrations.

Recently, a set of indicators that integrate the number of compounds detected per person and their corresponding concentrations was proposed, including the number of compounds detected at high concentrations. The analyses were based on the general population of Catalonia, Spain [12]. Because studies in the U.S. on combinations of POPs and other chemicals raised relevant questions about the levels and effects of such mixtures [8,9,16,40–44], and because of the relatively large size of the U.S. population, we aimed at applying the methodology [8] to the U.S. general population.

Therefore, the objectives of the present study were to analyze the number of POPs detected per person at high concentrations (nPhc) in the U.S. National Health and Nutrition Examination Survey (NHANES), and to analyze the associations between such indicator and main socioeconomic factors. Our main hypotheses were that most of the U.S. population would have POPs at low and high concentrations, and that sociodemographic factors (such as age, gender, body mass index (BMI), parity, or income) that are often related with POP concentrations when each compound is analyzed individually [2,12,13,30,34,35] would continue to show similar relationships when the POPs are jointly analyzed [12].

## Materials and Methods

### Data

Conducted by the Centers for Disease Control and Prevention’s (CDC) National Center for Health Statistics (NCHS), the National Health and Nutrition Examination Survey (NHANES) collects nationally representative environmental biomonitoring data from about 5,000 annual participants in each two-year cycle [2,45–48]. NHANES is a publicly available data set, and all participants provide written informed consent, consistent with approval by the NCHS Institutional Review Board. Ethical approval for use of NHANES data is not required as it is anonymized. We examined data from NHANES laboratory and demographic files corresponding to 2003–2004, which is the last period with valid updated individual data for compounds considered in the present study [45,47]. Except for perfluorinated compounds (PFCs), in NHANES 2005–2006 and 2007–2008 serum concentrations of POPs were measured using weighted pooled-samples, and no data for POPs have been published for NHANES 2009–2010 and 2011–2012 [47]. Therefore, it is not possible to calculate the number of POPs detected per person at high concentrations in more recent periods.

In each NHANES, most chemicals or their metabolites were measured in serum samples from random subsamples of about 2,500 participants aged 12 years and older. The chemicals’ concentrations were analyzed by CDC’s Environmental Health Laboratory using mass spectrometry and related methods [2,46]. Data for 91 POPs were analyzed, including: 13 organochlorine compounds (OCs) and their respective metabolites; 10 polybrominated diphenyl ethers (PBDEs); the polybrominated biphenyl (PBB) 153; 29 non-dioxin-like polychlorinated biphenyls (PCBs); 3 dioxin-like coplanar PCBs; 6 dioxin-like mono-ortho-substituted PCBs; 10 polychlorinated dibenzofurans (PCDFs); 7 polychlorinated dibenzo-p-dioxins (PCDDs); and 12 PFCs [46] (S1 Table). Thus, serum concentrations of lipophilic chemicals (e.g., dioxins and PCBs) are presented per gram of total lipid (better reflecting the amount stored in body fat) [1,2,12,31,46]; results of analyses per whole weight of serum were similar and are not presented. Concentrations of PFCs, non-lipophilic POPs, are shown per liter of serum. Limits of detection (LOD) for whole weight POP concentrations were different for each serum sample of each person [45,46], while LODs for lipid-adjusted concentrations were the same values for all samples and individuals (values ranged from 3.8 pg/g of lipid and 7.8 ng/g of lipid) [2,45,46]. Finally, LODs for PFCs ranged from 0.1 to 1.0 μg/L [46].

We considered important covariates as age, sex, race/ethnicity (non-Hispanic white, henceforth ‘White’; Mexican American; non-Hispanic black, henceforth ‘Black’; other Hispanic; and other), education (categorized to less than high school diploma, high school diploma, and greater than high school diploma), and body mass index (BMI) in kg/m^2^. To estimate the participants' income we used the family’s total income divided by the family size-specific poverty threshold income ratio (PIR), with two categories: “Low” income (PIR < 2), and “High” income (PIR ≥ 2) [43,45]. In women, we also considered the number of pregnancies resulting in live births, and the number of children breastfed ≥1 month (henceforth, ‘breastfeeding’) [45].

### Statistical analyses

The present study included 4,739 participants ≥20 years old (for all adults 85 years and older, age was coded at 85 years to reduce the risk of disclosure) [45]. They came from three subsamples (S2 Table). There were no significant differences between the 1,610, 1,585 and 1,544 participants of each subsample in a broad range of sociodemographic variables (including sex, race/ethnicity, educational level, PIR, BMI, number of pregnancies or breastfeeding) (S2 Table).

We imputed the unmeasured POP values by the median serum concentration of each POP according to age, sex, race/ethnicity, PIR, BMI, and, in women, number of pregnancies [49,50]. In 79 POPs the imputation was performed using concentrations adjusted by lipids, and in 12 PFCs, in μg/L. We calculated the total toxic equivalency (TEQ) [51,52] for 26 POPs: 3 dioxin-like coplanar PCBs, 6 dioxin-like mono-*ortho*-substituted PCBs, 10 PCDFs, and 7 PCDDs [45,46]. To compare POP concentrations in the present study and pooled concentrations in NHANES 2005–2006 and 2007–2008 we computed concentrations of POPs by sex, race/ethnicity and age groups [46,47]. We also compared PFC serum concentrations in the present study and concentrations in NHANES of 2005–2006, 2007–2008, 2009–2010 and 2011–2012 [46,47]. Descriptive values for POP concentrations imputed are summarized in S1 Table, sorted from the highest to the lowest percentage of detection.

Based on previous work by Porta et al. (2012) [12], we calculated the number of POPs detected in each person at high concentrations (nPhc) as follows: for each subject we added the number of POPs whose serum concentrations were equal to or greater than a selected cutoff point [12]. To be conservative, in the main analyses we included only 37 POPs that had been detected (each) in >85% of the study subjects (henceforth called the most prevalent POPs). Such 37 POPs were: 2 OCs, 3 PBDEs, PBB 153, 23 non-dioxin-like PCBs, 3 dioxin-like PCBs, one PCDD [1,2,3,4,6,7,8-Heptachlorodibenzo-*p*-dioxin (HpCDD)], and 4 PFCs (S1 Table). Ancillary analyses included 50 compounds detected in >50% of subjects. Finally, other analyses included all 91 POPs, with quartiles, quintiles and deciles defined after the imputation of concentrations (S1 Table) [12]. As usual, serum concentrations of POPs did not follow a normal distribution [31], and the increment of concentrations in the highest percentiles was very strong (e.g., for *p*,*p’*-DDE the increment of concentrations between P75 and P90 was of 2.14 times, and between P90 and the maximum it was 14.5 times; for PCB 153 the corresponding figures were 53% and 12.36 times, respectively) (S1 Table).

We defined ‘high concentrations’ using compound- and population-specific percentiles, based on actual POP distributions, as cutoff points [12,43,44]. In the main statistical analyses the cutoff point used was percentile 90 (P90), the upper decile (S1 Table).

Univariate statistics were computed as customary [53,54]. The highest correlations were observed between PCB congeners 170 and 180, 138 and 153, 146 and 153 (all Spearman’s *ρ* >0.982 and *p’s* <0.001). Fisher’s exact test for homogeneity was applied to assess the relationship between two categorical variables. For comparisons between continuous variables ANOVA, Kruskal-Wallis, and Mann-Whitney’s *U* tests were used. When a tendency was observed, Mantel–Haenszel’s χ^2^ test and Jonckheere-Terpstra test for linear trend were used.

To estimate the magnitude of associations between the socioeconomic factors and the number of most prevalent POPs with concentrations in the upper decile, multivariate-adjusted odds ratios (ORs) and their corresponding 95% confidence intervals (CI) were calculated by unconditional logistic regression with progressive degrees of adjustment [55]. The main effects of all predictors were independently explored in the base models, and final models were adjusted for age, gender, BMI, race/ethnicity and poverty income, in accordance with the nature of the variables and the study objectives. The number of POPs with concentrations in the upper decile was tested in different regression models using 3 different categorizations (all dichotomous): ≥1 POP (vs. no Phc), ≥6 POPs (vs. <6 POPs) and ≥10 POPs (vs. <10 POPs). Categorical ordinal variables were analyzed for a linear dose–response relation through the multivariate analogue of Mantel’s extension test; when a linear trend was not apparent, the probability test was used. Analyses were conducted using SPSS version 18 (SPSS, Armonk, NY, USA, 2009).

## Results

Over 67% of the 4,739 participants (73.8% of men and 61.1% of women) had one or more of the 37 most prevalent POPs at concentrations equal to or greater than the 90th percentile (≥P90), while 38.0% had ≥3 POPs, and over 13% had ≥10 POPs each in such top decile (Table 1 and Table 2). Over 37% of subjects had ≥10 compounds each at concentrations in the top quartile (≥P75) (S3 Table). The number of POPs detected per person ranged between 23 and 74, with an average of 49.7. Over 57% of participants had ≥50 POPs detected (S1 Fig).

**Table 1:** Characteristics of subjects with and without ten or more POPs with concentrations in the upper decile.

			≥10 POPs with concentrations in the upper decile				
Characteristics	Total		Yes		No		
	N (%)		N (%)		N (%)		*p*-value
**Total**	4,739	(100)	619	(13.1)	4,120	(86.9)	
**Gender**							<0.001
Women	2,467	(52.1)	271	(11.0)	2,196	(89.0)	
Men	2,272	(47.9)	348	(15.3)	1,924	(84.7)	
**Age** (years)	49.0		70.0		45.0		<0.001^a^
**Race/ethnicity**							<0.001
Non-Hispanic white	2,539	(53.6)	282	(11.1)	2,257	(88.9)	
Mexican American	951	(20.1)	21	(2.2)	930	(97.8)	
Non-Hispanic black	948	(20.0)	277	(29.2)	671	(70.8)	
Other Hispanic	140	(3.0)	19	(13.6)	121	(86.4)	
Other	161	(3.4)	20	(12.4)	141	(87.6)	
**Educational level**							<0.001
College or above	2,138	(45.2)	243	(11.4)	1,895	(88.6)	
High school	1,193	(25.2)	140	(11.7)	1,053	(88.3)	
< High school	1,399	(29.6)	232	(16.6)	1,167	(83.4)	
**Poverty income ratio**							0.036
>2	2,394	(53.6)	289	(12.1)	2,105	(87.9)	
≤2	2,075	(46.4)	295	(14.2)	1,780	(85.8)	
**Body mass index** (kg/m^2^)	27.4		26.7		27.6		0.004^a^
Underweight (<18.5)	70	(1.5)	10	(14.3)	60	(85.7)	<0.001^b^
Normal weight (18.5–24.9)	1,406	(30.3)	193	(13.7)	1,213	(86.3)	
Overweight (25.0–29.9)	1,630	(35.1)	248	(15.2)	1,382	(84.8)	
Obese (≥30)	1,538	(33.1)	154	(10.0)	1,384	(90.0)	
**Pregnancy**^**c**^							0.741
No	93	(5.0)	12	(12.9)	81	(87.1)	
Yes	1,768	(95.0)	208	(11.8)	1,560	(88.2)	
**No. of pregnancies resulting in live births**^**c**^	2.00		3.00		2.00		<0.001^a^
**Breastfeeding**^**d**^^,^^**e**^							0.450
No	78	(7.4)	6	(7.7)	72	(92.3)	
Yes	975	(92.6)	106	(10.9)	869	(89.1)	
**No. of children breastfed**^**d**^^,^^**e**^	2.00		2.00		2.00		0.144^a^

Values for age, body mass index, number of pregnancies resulting in live births and number of children breastfed are median.

Unless otherwise specified, *p*-Value derived from Fisher’s exact test (two-tail).

^a^ Mann-Whitney’s *U* test.

^b^ Without participants <18.5 kg/m^2^ of body mass index.

^c^ Women only.

^d^ Only women with ≥1 pregnancies resulting in live births.

^e^ Breastfed ≥1 month.

**Table 2:** Characteristics of the individuals with one or more POPs with concentrations in the upper decile.

			No. of POPs with concentrations in the upper decile										
Characteristics	Total		≥10		6 to 9		3 to 5		2		1		
	N (%)		N (%)		N (%)		N (%)		N (%)		N (%)		*p*-value
**Total**	3,184	(67.2)	619	(19.4)	375	(11.8)	807	(25.3)	560	(17.6)	823	(25.8)	
(Cumulative %)				(19.4)		(31.2)		(56.6)		(74.2)		(100)	
**Gender**													<0.001
Women	1,507	(47.3)	271	(18.0)	135	(9.0)	406	(26.9)	272	(18.0)	423	(28.1)	
Men	1,677	(52.7)	348	(20.8)	240	(14.3)	401	(23.9)	288	(17.2)	400	(23.9)	
**Age** (years)	57.0		70.0		60.0		64.0		46.0		43.0		<0.001^a^
**Race/ethnicity**													<0.001^b^
Non-Hispanic white	1,780	(55.9)	282	(15.8)	233	(13.1)	530	(29.8)	270	(15.2)	465	(26.1)	
Mexican American	581	(18.2)	21	(3.6)	76	(13.1)	159	(27.4)	154	(26.5)	171	(29.4)	
Non-Hispanic black	629	(19.8)	277	(44.0)	40	(6.4)	76	(12.1)	93	(14.8)	143	(22.7)	
Other Hispanic	79	(2.5)	19	(24.1)	11	(13.9)	17	(21.5)	16	(20.3)	16	(20.3)	
Other	115	(3.6)	20	(17.4)	15	(13.0)	25	(21.7)	27	(23.5)	28	(24.3)	
**Educational level**													<0.001
College or above	1,403	(44.2)	243	(17.3)	168	(12.0)	339	(24.2)	248	(17.7)	405	(28.9)	
High school	790	(24.9)	140	(17.7)	103	(13.0)	210	(26.6)	144	(18.2)	193	(24.4)	
< High school	984	(31.0)	232	(23.6)	103	(10.5)	256	(26.0)	168	(17.1)	225	(22.9)	
**Poverty income ratio**													0.002
>2	1,608	(53.4)	289	(18.0)	189	(11.8)	389	(24.2)	295	(18.3)	446	(27.7)	
≤2	1,406	(46.6)	295	(21.0)	175	(12.4)	365	(26.0)	240	(17.1)	331	(23.5)	
**Body mass index** (kg/m^2^)	27.2		26.7		28.1		26.8		27.6		27.8		0.084^a^
**Pregnancy**^**c**^													0.447^b^
No	51	(4.4)	12	(23.5)	4	(7.8)	9	(17.6)	8	(15.7)	18	(35.3)	
Yes	1,106	(95.6)	208	(18.8)	97	(8.8)	306	(27.7)	193	(17.5)	302	(27.3)	
**No. of pregnancies resulting in live births**^**c**^	3.00		3.00		2.00		3.00		3.00		2.00		<0.001^a^
**Breastfeeding**^**d**^^,^^**e**^													0.067
No	47	(7.4)	6	(12.8)	3	(6.4)	11	(23.4)	7	(14.9)	20	(42.6)	
Yes	592	(92.6)	106	(17.9)	42	(7.1)	180	(30.4)	104	(17.6)	160	(27.0)	
**No. of children breastfed**^**d**^^,^^**e**^	2.00		2.00		2.00		2.00		2.00		2.00		0.001^a^

Values for age, body mass index, number of pregnancies resulting in live births and number of children breastfed are median.

Unless otherwise specified, *p*-Value derived from Mantel–Haenszel’s χ^2^ test for linear trend.

^a^ Jonckheere-Terpstra test for linear trend.

^b^ Fisher’s exact test (two-tail).

^c^ Women only.

^d^ Only women with ≥1 pregnancies resulting in live births.

^e^ Breastfed ≥1 month.

In over 45% of participants who had only one POP at high concentrations (Phc) (≥P90), this chemical was an OC, a PBDE or PBB 153. By contrast, among subjects with numerous Phc, the majority of such compounds were PCBs. For instance, when the nPhc was ≥3, more than 40% of these compounds were PCBs and HpCDD.

The median age of participants with ≥10 POPs at high concentrations (Phc) was 70 years, while for participants with <10 Phc it was 45 years, and for participants without any Phc, 39 years. Over 11% of Whites, 2.2% of Mexican Americans, and 29.2% of Blacks had ≥10 Phc (*p* <0.001) (Table 1). Subjects with ≥10 Phc had a slightly lower median BMI than subjects with <10 Phc (26.7 Kg/m^2^ and 27.6 Kg/m^2^, respectively, *p* for trend = 0.004) (Table 1). Women with ≥10 Phc had a higher number of pregnancies resulting in live births than women with <10 Phc (age-unadjusted medians: 3.0 and 2.0, respectively, *p* for trend <0.001). There were significant differences in the nPhc by sex, age, BMI, race/ethnicity, educational level, PIR, and, in women, by number of pregnancies, and breastfeeding (Table 2).

**Fig 1:**
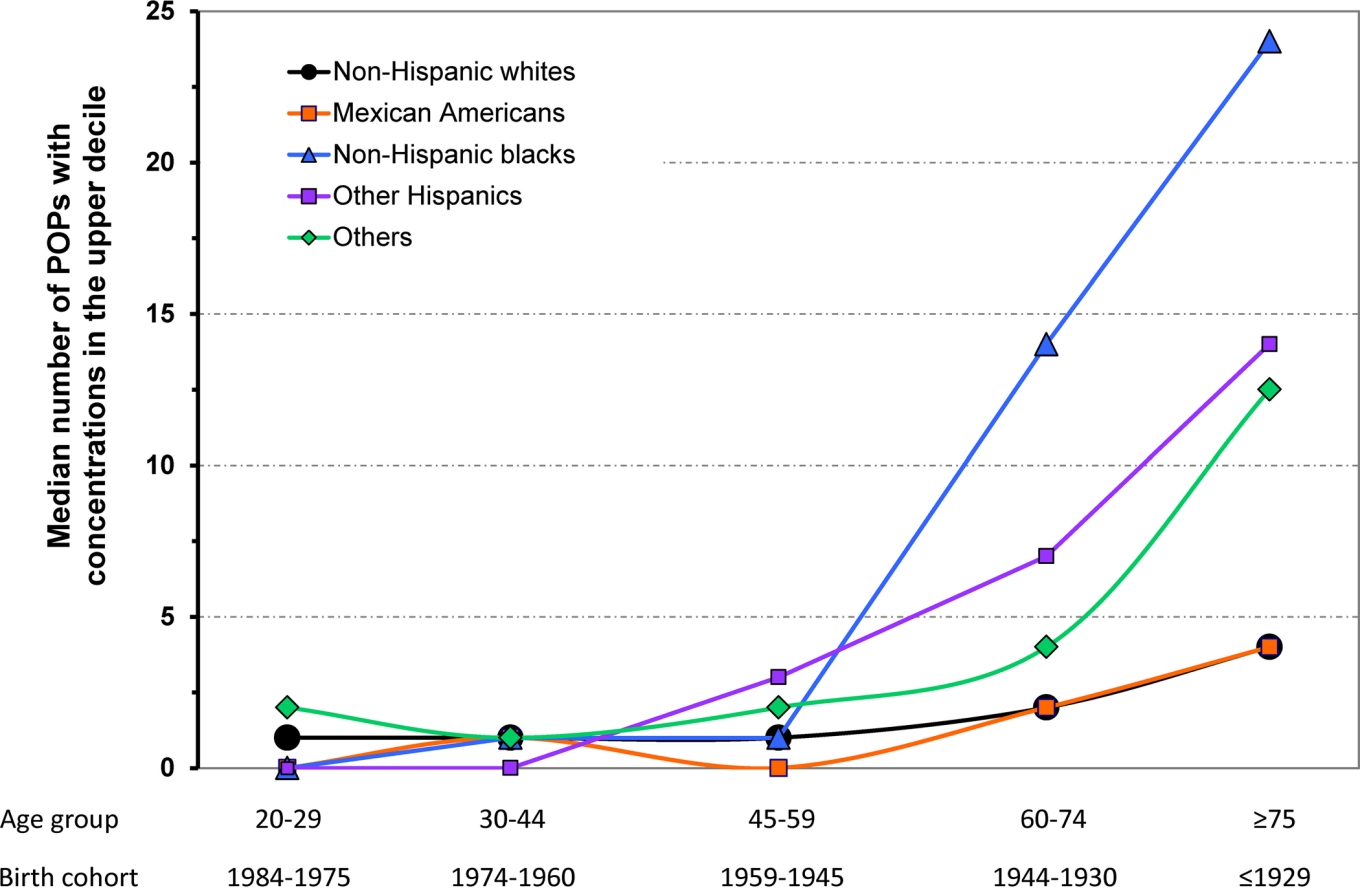
Median number of POPs with concentrations in the upper decile by age/birth cohort and race/ethnicity.

Multivariate analyses adjusted by age, gender and BMI showed that, as compared to Whites, Blacks had an odds ratio (OR) = 10.1 of having ≥10 Phc, whilst for Mexican Americans the OR was 0.2 (both *p’s* <0.001) (Table 3). When further adjusted by poverty income the OR for Blacks decreased to 9.2, and for Mexican Americans to 0.18 (both *p’s* <0.001). Differences between Blacks and Whites were larger in the older age groups / birth cohorts, and null in the younger ones (*p* for interaction <0.001) (Fig 1).

**Table 3:** Associations between sociodemographic characteristics and having ten or more POPs with concentrations in the upper decile.

	Model 1			Model 2			Model 3		
Characteristics	OR^a^	(95% CI)		OR^a^	(95% CI)		OR^a^	(95% CI)	
**Gender**									
Women	1.00			1.00			1.00		
Men	1.54***	(1.28,	1.85)	1.74***	(1.42,	2.13)	1.73***	(1.40,	2.13)
**Age** (years)	1.06***	(1.05,	1.07)	1.08***	(1.07,	1.09)	1.08***	(1.07,	1.09)
**Race/ethnicity**									
Non-Hispanic white	1.00			—			1.00		
Mexican American	0.22***	(0.14,	0.35)				0.18***	(0.11,	0.30)
Non-Hispanic black	10.11***	(7.82,	13.1)				9.18***	(7.05,	12.0)
Other Hispanic	3.17***	(1.78,	5.63)				2.64**	(1.43,	4.85)
Other	1.94*	(1.11,	3.37)				1.55	(0.84,	2.88)
**Educational level**									
College or above	1.00			1.00			1.00		
High school	0.91	(0.72,	1.15)	0.96	(0.75,	1.24)	0.96	(0.74,	1.25)
< High school	1.04	(0.84,	1.28)	1.22	(0.95,	1.55)	1.14	(0.88,	1.49)
**Poverty income ratio**									
>2	1.00			1.00			—		
≤2	1.13	(0.94,	1.37)	1.24*	(1.00,	1.53)			
**Body mass index** (kg/m^2^)									
Normal weight	1.00			1.00			1.00		
Overweight	0.96	(0.77,	1.20)	0.96	(0.75,	1.22)	1.02	(0.79,	1.30)
Obese	0.74*	(0.58,	0.94)	0.57***	(0.44,	0.75)	0.58***	(0.44,	0.77)
**Pregnancy**^b^									
No	1.00			1.00			1.00		
Yes	0.48*	(0.24,	0.99)	0.57	(0.24,	1.38)	0.60	(0.25,	1.46)
**No. of pregnancies resulting in live births**^b^	0.99	(0.92,	1.06)	0.97	(0.89,	1.06)	0.97	(0.88,	1.06)
**Breastfeeding**^c^^,^^d^									
No	1.00			1.00			1.00		
Yes	1.15	(0.46,	2.87)	1.36	(0.48,	3.84)	1.30	(0.46,	3.68)
**No. of children breastfed**^c^^,^^d^	0.88	(0.76,	1.01)	0.97	(0.82,	1.14)	0.96	(0.81,	1.13)

Model 1: adjusted by age, gender and body mass index.

Model 2: adjusted by age, gender, body mass index and race/ethnicity.

Model 3: adjusted by age, gender, body mass index, race/ethnicity and poverty income.

^a^ *p*-Value derived from Wald’s test.

^b^ Women only.

^c^ Only among women with ≥1 pregnancies resulting in live births and, in the three models, further adjusted by such number of pregnancies.

^d^ Breastfed ≥1 month.

* *p* ≤0.05

** *p* ≤0.01

*** *p* ≤0.001.

For PIR ≤2, or “Low” income (vs. PIR>2 or “High” income) the OR of having ≥10 Phc was 1.13 (*p* >0.05) when the model was adjusted by age, gender and BMI, and 1.24 (*p* = 0.045) when further adjusted by race/ethnicity. The OR for obesity (vs. normal weight) was 0.74 (*p* for trend = 0.015) in the model adjusted by age and gender, and, when further adjusting by race/ethnicity and poverty income, it was 0.58 (*p* for trend <0.001) (Table 3). In women, pregnancy halved the probability of having ≥10 Phc when adjusting by age and body mass index (OR = 0.48, *p* = 0.048) (Table 3).

In models assessing the relationship between sociodemographic factors and the probability of having ≥1 POPs at concentrations ≥P90 (vs. not having POPs with concentrations ≥P90), adjusted by age, gender and BMI, the OR for Blacks (vs. Whites) was 1.10 (*p* = 0.266) and for Mexican Americans, 0.74 (*p* <0.001). When further adjusted by race/ethnicity, the OR for PIR≤2 (vs. PIR>2) was 1.17 (*p* = 0.026); and for obesity (vs. normal weight), 0.73 (*p* for trend <0.001 for BMI) (S4A Table). The corresponding figures for the probability of having ≥6 POPs at concentrations ≥P90 were 3.37 for Blacks and 0.51 for Mexican Americans (both *p* <0.001), 1.24 for PIR≤2 (*p* <0.01), and 0.91 for obesity (vs. normal weight) (*p* for trend = 0.353).

Because of the influence of PCBs in the previous results, we also analyzed associations among sociodemographic factors and the likelihood of having ≥1 of 6 POPs other than PCBs (i.e., OCs, PBDEs, and PBB 153 detected ≥85% of subjects) at high concentrations (S4B Table). Contrary to what was observed when all compound families were considered, for Blacks (vs. Whites) the OR of having ≥1 of such POPs was 0.76, and for Mexican Americans, 1.41, adjusting by age, gender and BMI (both *p’s* <0.01). The corresponding OR for PIR≤2 was 1.19 (*p* = 0.011), and for obesity, 0.82 (*p* for trend = 0.013). When further adjusting by race/ethnicity, the OR for PIR≤2 was 1.15 (*p* = 0.038), and for obesity, 0.80 (*p* for trend = 0.006) (S4B Table).

The geometric mean (GM) of nPhc doubled when the cutoff P75 was used instead of P90 (see S3 Table). For the cutoff P75 the percentage of subjects with ≥10 Phc was 37.5 for POPs detected in ≥85% of participants, and 45.2 for POPs detected in ≥50% of participants. However, the percentage of subjects without any Phc decreased slightly when the number of POPs included in the analyses increased.

In the 1,183 participants with the highest total TEQ concentrations (≥P75 of the distribution of total TEQ concentrations [i.e., ≥26.68 pg WHO-TEQ/g of lipid]), the percentage of subjects with ≥10 Phc was about twice the corresponding figure observed when all 4,739 participants were considered. Over 90% of the 1,183 subjects had ≥1 POPs a) not included in TEQ calculations, and b) with concentrations ≥P90. Over 30% had ≥10 such POPs, and almost 7% had ≥20 such POPs. Spearman’s *ρ* coefficient between the total TEQ concentration and nPhc (considering the 24 POPs not included in TEQ calculations, and the P90 in all participants for high concentrations) was 0.475 (*p* <0.001).

Over 43% of participants had TEQ concentrations ≥21 pg WHO-TEQ/g of lipid, a biomonitoring equivalent value (see Discussion). Taking into account health-based guidelines for other compounds, less than 1% of participants had concentrations of hexachlorobenzene ≥47 ng/g of lipid, and concentrations for the sum of *p*,*p’*-DDT and *p*,*p’*-DDE ≥5,000 ng/g of lipid. Two subjects had concentrations of BDE 99 ≥520 ng/g of lipid; 2 participants (aged <40 years) had concentrations ≥700 ng/g of lipid for the sum of 35 PCBs (without dioxin-like coplanar PCBs), and 6 participants (aged ≥40 years) had concentrations ≥1,800 ng/g of lipid. 10% of participants had concentrations for the sum of PCBs 138, 153 and 180 ≥216 ng/g of lipid. Only 4 participants had concentrations for the sum of these three PCBs ≥900 ng/g of lipid.

We also compared the concentrations of POPs detected in ≥85% of the participants in the present study (2003–2004), and their respective pooled concentrations for the NHANES periods 2005–2006 and 2007–2008. The concentrations of some POPs in the present study were only slightly higher than in subsequent periods; they were not higher or not statistically significant in the case of *p*,*p’*-DDE, PBB 153 and some PBDEs compounds (S5A Table). For 3 PFCs, concentrations in 2003–2004 were similar to concentrations in 2005–2010, and slightly higher than concentrations in 2011–2012 (S5B Table).

## Discussion

More than half of the study population had concentrations in the top decile of ≥1 of the most commonly detected POPs, 38% had ≥3, and over 13% had ≥10 POPs each in their respective top decile. Findings are thus partly in contrast with the notion that human POP concentrations are low in the vast majority of the population [5,12]: such view holds only when each individual compound is looked at separately, but not when the individual human is of concern.

Median age of participants with ≥10 of most prevalent POPs at high concentrations was 70 years, while median age of participants without any Phc was 39 years. This could be due to biological aging effects or to birth cohort effects. Furthermore, the median age of participants without any Phc was near the median age of participants with 1 or 2 Phc. There were also significant differences in the nPhc by gender, race/ethnicity, educational level, PIR, BMI, parity, and breastfeeding. These results are in accordance with our main hypotheses (most of the U.S. population had POPs at low and high concentrations; sociodemographic factors related with each POP concentration showed similar relationships for the joint analysis of POPs).

Race/ethnicity was the sociodemographic factor most associated with a higher nPhc: Blacks had 9 times a greater chance of having ≥10 Phc than Whites, and Mexican Americans over 4 times a lower chance. The nPhc indicator not only shows that Blacks have higher body concentrations of POPs than Whites (or Mexican Americans lower concentrations), but it also quantifies how many POPs are in a specific high concentration range. The NHANES questionnaires had a large number of sociodemographic items; in this study, we used the sociodemographic factors that were available and related with body concentrations of POPs [12,32,35,39,45,46].

Results of unconditional logistic regression models for ≥1 Phc, ≥6 Phc, and ≥10 Phc (vs. no Phc, <6 Phc, and <10 Phc, respectively) in the subsample without imputations and PCBs, PCDDs/Fs analyzed by the sociodemographic factors (to assess the possible biases of imputations), were similar to results of models with imputations, except in some models for gender, which was not statistically significant, although ORs were similar.

Most studies found an inverse association between PCB levels in blood and BMI [56–58], as in the present analyses for all participants and 37 POPs.

Also rarely if ever noted before: high percentages of subjects with TEQ ≥P75 (≥26.68 pg WHO-TEQ/g of lipid) had numerous POPs not included in TEQ calculations, at high concentrations. Findings suggest that studies using TEQ measures could be even more relevant if they additionally assessed subgroups with high nPhc. Results do not imply that nPhc and related exposure indicators are preferable to other indicators to evaluate associations between POP mixtures and clinical outcomes; nPhc indicators just provide a different and complementary approach to indicators such as the sum of concentrations of PCBs [48,59–64], or the sum of orders of POPs [65].

Our goal was not to evaluate whether individuals have increased health risks due to multiple compounds at high concentrations, nor to assess the role of modes and mechanisms of action, but to propose a new and useful approach for exposure assessment. However, severe adverse health effects have been reported for concentrations similar to or lower than P90 in the present study [5,9,11,15,16,66–69]; e.g., in an NHANES study the OR of having diabetes for a concentration of ≥60.2 ng/g lipid of PCB 153 was 5.9 (95% CI = 3.0–11.9) [66]; in the present study the P90 for PCB 153 was 79.8 ng/g lipid. P90 of concentrations of individual PCBs in NHANES is as high or higher than in other countries with population-based surveys as Canada and Australia [6,70]. For *p*,*p’*-DDE and *β*-hexachlorocyclohexane (*β*-HCH), it is also as high or higher than in Canada, Australia and Germany [6,70].

In this study, over 43% of participants had TEQ concentrations ≥21 pg WHO-TEQ/g of lipid, which is the biomonitoring equivalent value published for dioxin TEQ, a health-risk based screening guideline [71]. Also, in the present study 10% of participants had concentrations for the sum of PCBs 138, 153 and 180 equal to or greater than the Human Biomonitoring level-I (HBM-I), which is 3 μg/L plasma or, when accounting for lipids, 216 ng/g lipid for the present study. HBM-I is a health-related exposure limit recommended for PCBs by the German Human Biomonitoring Commission [59–62]. For compounds considered in the present study, other biomonitoring equivalents values are only available for hexachlorobenzene, the arithmetic sum of *p*,*p’*-DDT and *p*,*p’*-DDE, the sum of 35 PCBs, and BDE 99 [59,71,72]; for these compounds very few subjects had concentrations above the corresponding biomonitoring equivalents in this study. To our knowledge, no current health-related limit values are available for the rest of PBDEs or for PFCs [59–61,71,72]. Although there are regulations and guidelines for other pollutants (e.g. lead, mercury, cadmium and other metals) and for POPs in air, soil, water and food (e.g., tolerable daily intakes), there are hardly any other guidelines for human POP concentrations to define levels of concern than the ones mentioned above [59–62,71].

Beyond findings on concentrations of individual compounds, the indicators illuminate a crucial–and usually overlooked–feature of human contamination by POPs: the frequency of mixtures of POPs at high concentrations. The approach could naturally be developed to integrate other pollutants of concern.

Importantly, 2003–2004 is the last period of NHANES in which the individual concentration of each compound is available for each individual subject. In 2005–2006 and 2007–2008 serum concentrations of POPs (except PFCs) were measured in weighted pooled-samples (not in individual samples); no data were published for 2009–2010 and 2011–2012. Therefore, data to calculate the number of POPs detected per person at high concentrations in more recent periods are not available.

Concentrations of most POPs in 2003–2004 were only slightly higher than in more recent periods (Tables in S5 File). Virtually all major contemporary health effects of POPs will be influenced by concentrations experienced by human cohorts during several decades, not just by recent exposures. Furthermore, the nPhc can be fruitfully applied to analyze data from many periods and settings.

Different POPs were analyzed in participants of the three NHANES 2003–2004 subsamples; even in two of the three subsamples all selected POPs were not analyzed in all participants. Each sample, however, is valid; and it is efficient not to analyze all POPs in all participants [2,46]. For PFCs, the LODs were constant for each sample analyzed [46]. For the other 55 compounds, the LOD for the whole weight concentrations was different for each serum sample of each person [2]. When PCDD/Fs, PCBs, OCs, PBDEs, and PBB 153 concentrations were measured in serum lipid, LOD calculations were performed using the chemical concentration expressed per amount of lipid, and the LOD concentration expressed per amount of lipid was the highest LOD among all the individual samples analyzed [2,46]. LODs for lipid adjusted concentrations were highest compared to the LODs for the whole weight concentrations, and rates of detection were lower, than for whole weight concentrations; as a consequence, lipid adjusted results are more conservative (e.g., because there were less compounds detected in ≥85% of participants).

In the present study, some associations between nPhc and sociodemographic factors are quite influenced by the predominance of PCBs and HpCDD at high concentrations among subjects with ≥3 nPhc. The cutoff point for nPhc should be chosen with this issue in mind, while also avoiding a too high nPhc (e.g., because of lower detection rates of some POPs) [12].

Serum concentrations of POPs do not follow a normal distribution [31]. Values for P90 can be much higher than the P75 (e.g., for *p*,*p’*-DDE the P90 value was 2.14 times greater than P75, and for PCB 153 it was 53%). Such differences between highest concentrations minimize a possible misclassification of concentrations in ≥P90 or <P90 due to laboratory measurement errors [46]. The minimum percentage of participants with concentrations in the top decile of ≥1 POPs will be 10%, but such percentage will not necessarily, linearly, or indefinitely increase (nor approach 100%) as the number of compounds considered increases: the percentage of participants with concentrations of ≥1 POPs in the top decile is only partly positively influenced by the number of compounds considered; it is also inversely influenced by the magnitude of the correlations between the pairs of compounds, being highest when POPs are completely uncorrelated (for details see Suppl. Material of Porta et al., 2012) [12]. Therefore, the nPhc follows a distribution that is influenced by all the correlations between the pairs of compounds, and results may not be due to chance. Figure 1 of Supplemental Material of Porta et al., 2012 [12] shows different values for ≥1 by the number of POPs considered, and the values when the POPs were completely uncorrelated. For ≥10 POPs at high concentrations (rather than ≥1 POP) this situation is even more restrictive; when we focused on ≥10 POPs at high concentrations, it was statistically possible for the minimum percentage of participants with ≥10 POPs at high concentrations to be 0% (i.e., it was not statistically inevitable for that percentage to be 10%), since it is possible that highly-correlated sets of POPs comprise 9 or less POPs. Furthermore, such minimum percentage also depends on the number of POPs analyzed, the number of POPs in the top decile, and the number of participants included.

## Conclusion

In summary, more than 13% of the US population may have ≥10 POPs each at concentrations in the top decile. This finding is not to be expected just on statistical grounds. High percentages of subjects with TEQ ≥P75 have numerous POPs not included in TEQ calculations, at high concentrations. The nPhc is related to race/ethnicity, age, and BMI. It is also likely to be related to other relevant social, environmental, and individual factors. The study findings foster knowledge on previously unknown characteristics of human chemical contamination in the US population. Such knowledge is a right of citizens, and could also be considered when evaluating the impacts of relevant public and private policies.

## Supporting Information

S1 Fig Percentage of participants according to number of POPs detected per person. (91 POPs analyzed, n = 4,739)
(PDF)

S1 Table Limit of detection (LOD) and statistics of concentrations of 91 POPs for the 4,739 participants. (DOCX)

S2 Table Population characteristics by survey subsample. (DOCX)

S3 Table Frequency of subjects with high concentrations of the most detected POPs according to different definitions of ‘high concentration’. (DOCX)

S4 Table S4ATable. Associations between sociodemographic characteristics and having one or more POPs with concentrations in the upper decile. S4B Table. Associations between sociodemographic characteristics and having one or more OCs, PBDEs and PBB 153 with concentrations in the upper decile.
(DOCX)

S5 Table S5A Table. Serum pooled concentrations of POPs most detected for the three most recent NHANES Surveys periods analyzed. S5B Table. Serum concentrations of four perfluorinated compounds (μg/L) most detected for the most recent NHANES Surveys periods analyzed.
(DOCX)

## Abbreviations

*β*-HCH: *β*-hexachlorocyclohexane
BMI: body mass index
CDC: Centers for Disease Control and Prevention
CI: confidence interval
GM: geometric mean
HBM: Human Biomonitoring
LOD: limit of detection
NHANES: National Health and Nutrition Examination Survey
nPhc: number of POPs detected per person at high concentrations
PBBs: polybrominated biphenyls
PBDEs: polybrominated diphenyl ethers
PCBs: polychlorinated biphenyls
PCDDs: polychlorinated dibenzo-*p*-dioxins
PCDFs: polychlorinated dibenzofurans
PFCs: perfluorinated compounds
Phc: POPs at high concentrations
PIR: family’s total income divided by the family size-specific poverty threshold income
POP: persistent organic pollutant
OCs: organochlorine compounds
OR: odds ratio
TEQ: total toxic equivalency
