# Supplementary material for: Number of Persistent Organic Pollutants Detected at High Concentrations in Blood Samples of the United States Population
Source: PLoS One. 2016 Aug 10;11(8):e0160432. doi: 10.1371/journal.pone.0160432 (PMC4979965; doi:10.1371/journal.pone.0160432)
Supplement: S1 Fig — (91 POPs analyzed, n = 4,739) (PDF) [file pone.0160432.s001.pdf]

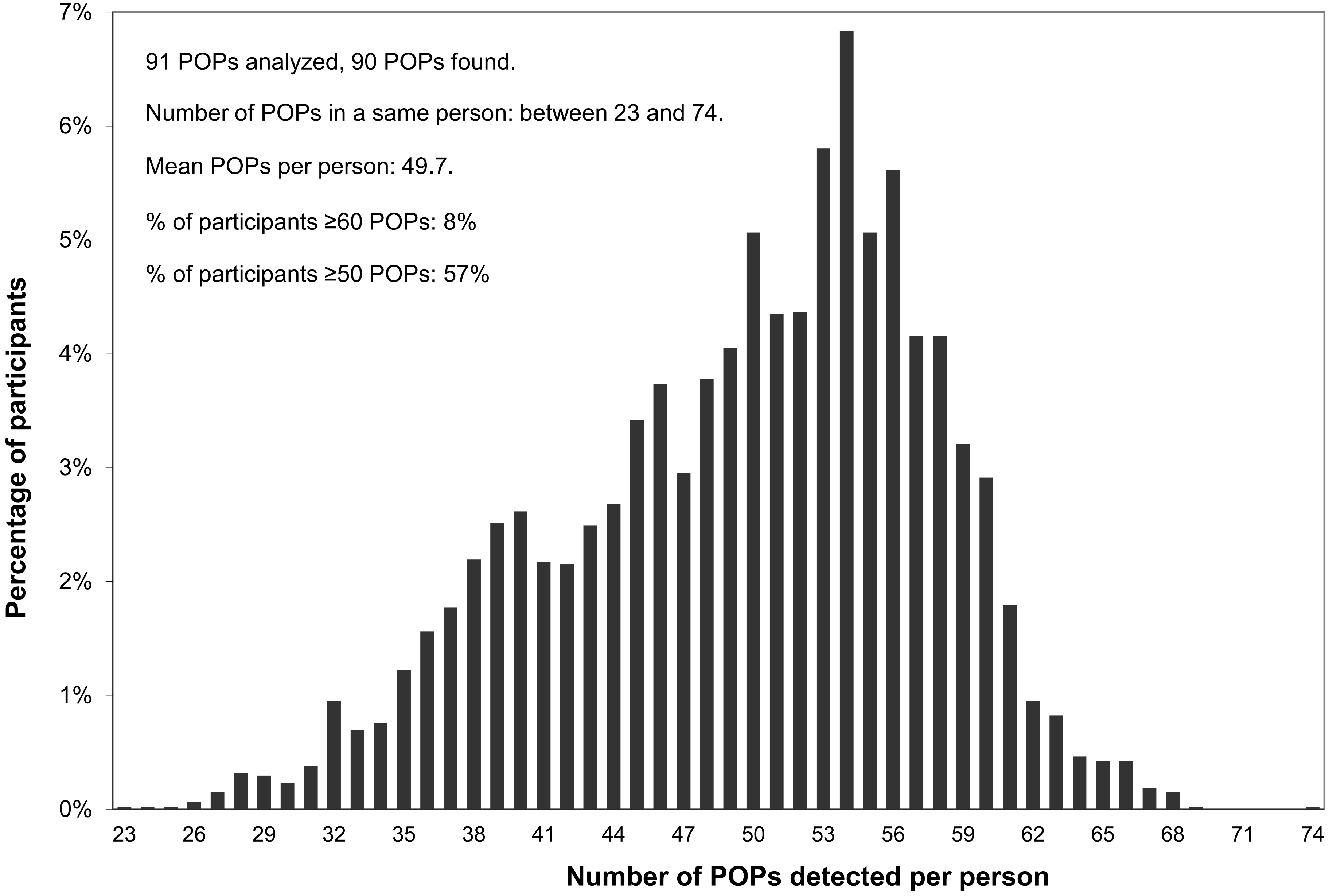

S1 Figure. Percentage of participants according to number of POPs detected per person.
(91 POPs analyzed, n = 4,739)
