# Supplementary material for: Number of Persistent Organic Pollutants Detected at High Concentrations in Blood Samples of the United States Population
Source: PLoS One. 2016 Aug 10;11(8):e0160432. doi: 10.1371/journal.pone.0160432 (PMC4979965; doi:10.1371/journal.pone.0160432)
Supplement: S1 Table — (DOCX) [file pone.0160432.s002.docx]

**S1 Table. Limit of detection (LOD) and statistics of concentrations of 91 POPs for the 4,739 participants.**

| **Persistent organic pollutant** | **Units of measurement** | **% ≥LOD** | **median** | **P75** | **P90** | **maximum** |
| --- | --- | --- | --- | --- | --- | --- |
| 2,2',3,4,4',5' and 2,3,3',4,4',6-Hexachlorobiphenyl (PCB 138 & 158) | ng/g of lipid | 100 | 23.50 | 36.30 | 58.90 | 773.0 |
| 2,3',4,4',5-Pentachlorobiphenyl (PCB 118) | ng/g of lipid | 100 | 6.80 | 12.96 | 25.00 | 354.0 |
| 2,2',4,4',5,5'-Hexachlorobiphenyl (PCB 153) | ng/g of lipid | 99.9 | 29.77 | 51.90 | 79.76 | 986.0 |
| 2,2',3,4,4',5,5'-Heptachlorobiphenyl (PCB 180) | ng/g of lipid | 99.9 | 25.80 | 45.18 | 67.17 | 627.0 |
| 2,2',3,5'-Tetrachlorobiphenyl (PCB 44) | ng/g of lipid | 99.9 | 1.94 | 2.40 | 3.30 | 85.40 |
| 2,4,4',5-Tetrachlorobiphenyl (PCB 74) | ng/g of lipid | 99.9 | 5.50 | 11.60 | 19.22 | 180.0 |
| *p,p'*-Dichlorodiphenyldichloroethene (DDE) | ng/g of lipid | 99.9 | 370.0 | 737.0 | 1580.0 | 22900 |
| 2,2',4,4',5-Pentachlorobiphenyl (PCB 99) | ng/g of lipid | 99.8 | 4.80 | 7.07 | 12.77 | 304.0 |
| Perfluorooctane sulfonic acid (PFOS) | μg/L | 99.8 | 20.50 | 25.50 | 35.20 | 435.0 |
| Perfluorooctanoic acid (PFOA) | μg/L | 99.8 | 3.65 | 4.70 | 6.20 | 77.20 |
| 2,2',3,3',4,4',5-Heptachlorobiphenyl (PCB 170) | ng/g of lipid | 99.7 | 9.30 | 15.63 | 22.18 | 299.0 |
| 2,2',3,4',5,5',6-Heptachlorobiphenyl (PCB 187) | ng/g of lipid | 99.6 | 6.86 | 12.50 | 18.95 | 158.0 |
| Perfluorononanoic acid (PFNA) | μg/L | 99.4 | 0.90 | 1.25 | 1.75 | 11.50 |
| Perfluorohexane sulfonic acid (PFHxS) | μg/L | 99.1 | 1.70 | 2.20 | 3.70 | 27.10 |
| 2,4,4'-Trichlorobiphenyl (PCB 28) | ng/g of lipid | 99.1 | 5.02 | 5.53 | 7.66 | 237.0 |
| 2,2',4,5'-Tetrachlorobiphenyl (PCB 49) | ng/g of lipid | 98.7 | 1.30 | 1.60 | 2.10 | 54.50 |
| 2,2',3,4',5,5'-Hexachlorobiphenyl (PCB 146) | ng/g of lipid | 98.6 | 3.50 | 5.84 | 9.23 | 86.75 |
| Hexachlorobenzene | ng/g of lipid | 98.5 | 15.90 | 19.00 | 22.45 | 174.0 |
| 2,2',5,5'-Tetrachlorobiphenyl (PCB 52) | ng/g of lipid | 98.0 | 2.70 | 3.30 | 4.55 | 133.0 |
| 2,2',3,3',4,4',5,6' and 2,2',3,4,4',5,5',6-Octachlorobiphenyl (PCB 196 & 203) | ng/g of lipid | 97.6 | 4.57 | 8.00 | 12.04 | 148.0 |
| 2,3,3',4,4'-Pentachlorobiphenyl (PCB 105) | ng/g of lipid | 97.3 | 1.32 | 2.40 | 4.24 | 76.82 |
| 2,2',4,4'-Tetrabromodiphenyl ether (BDE 47) | ng/g of lipid | 97.1 | 19.70 | 26.80 | 56.00 | 2350.0 |
| 2,3,3',4,4',5-Hexachlorobiphenyl (PCB 156) | ng/g of lipid | 96.2 | 4.56 | 7.76 | 11.71 | 367.0 |
| 2,2',3,3',4,5,5',6-Octachlorobiphenyl (PCB 199) | ng/g of lipid | 96.2 | 5.63 | 10.50 | 16.46 | 298.0 |
| 2,2',3,4,4',5',6-Heptachlorobiphenyl (PCB 183) | ng/g of lipid | 96.0 | 2.30 | 4.00 | 6.29 | 50.74 |
| 2,2',4,5,5'-Pentachlorobiphenyl (PCB 101) | ng/g of lipid | 95.4 | 1.64 | 2.15 | 3.20 | 79.30 |

[ Continued next page ]

**S1 Table, continued.**

| **Persistent organic pollutant** | **Units of measurement** | **% ≥LOD** | **median** | **P75** | **P90** | **Maximum** |
| --- | --- | --- | --- | --- | --- | --- |
| 2,2',3,3',4,4',5,5'-Octachlorobiphenyl (PCB 194) | ng/g of lipid | 94.7 | 5.61 | 10.35 | 16.57 | 149.0 |
| 2,3',4,4'-Tetrachlorobiphenyl (PCB 66) | ng/g of lipid | 93.2 | 1.40 | 1.90 | 2.55 | 118.0 |
| 2,2',3,3',4,5',6'-Heptachlorobiphenyl (PCB 177) | ng/g of lipid | 92.8 | 1.95 | 3.70 | 5.70 | 51.87 |
| 1,2,3,4,6,7,8-Heptachlorodibenzo-*p*-dioxin (HpCDD) | pg/g of lipid | 92.5 | 29.35 | 50.10 | 65.55 | 456.0 |
| 2,2',4,4',6-Pentabromodiphenyl ether (BDE 100) | ng/g of lipid | 91.3 | 3.50 | 5.20 | 10.50 | 339.0 |
| 2,2',3,4,5'-Pentachlorobiphenyl (PCB 87) | ng/g of lipid | 91.2 | 0.90 | 1.10 | 1.64 | 39.00 |
| 2,2',3,4',5',6-Hexachlorobiphenyl (PCB 149) | ng/g of lipid | 89.6 | 0.60 | 0.80 | 1.13 | 23.90 |
| 2,2',4,4',5,5'-Hexabromodiphenyl ether (BDE 153) | ng/g of lipid | 88.1 | 4.40 | 6.60 | 16.10 | 821.0 |
| 2,2',4,4',5,5'-Hexabromobiphenyl (BB 153) | ng/g of lipid | 87.7 | 2.70 | 4.10 | 8.10 | 225.0 |
| 2,3,3',4',6-Pentachlorobiphenyl (PCB 110) | ng/g of lipid | 86.8 | 1.20 | 1.51 | 2.27 | 59.20 |
| 2,2',3,3',4,4',5,5',6-Nonachlorobiphenyl (PCB 206) | ng/g of lipid | 85.9 | 3.30 | 7.01 | 11.80 | 176.0 |
| 2,2',3,3',5,5',6-Heptachlorobiphenyl (PCB 178) | ng/g of lipid | 82.8 | 1.72 | 2.95 | 4.68 | 29.76 |
| *trans*-Nonachlor | ng/g of lipid | 82.1 | 22.70 | 40.40 | 54.25 | 355.0 |
| 2,4,4'-Tribromodiphenyl ether (BDE 28) | ng/g of lipid | 81.4 | 1.20 | 1.65 | 3.20 | 65.20 |
| 1,2,3,6,7,8-Hexachlorodibenzo-*p*-dioxin (HxCDD) | pg/g of lipid | 79.0 | 27.20 | 43.50 | 54.90 | 206.0 |
| 2,2',3,3',4,5,5'-Heptachlorobiphenyl (PCB 172) | ng/g of lipid | 78.2 | 1.30 | 2.23 | 3.10 | 26.30 |
| 2,2',3,3',4,4',5,5',6,6'-Decachlorobiphenyl (PCB 209) | ng/g of lipid | 76.9 | 1.86 | 5.05 | 10.20 | 100.0 |
| 2,3,3',4,4',5'-Hexachlorobiphenyl (PCB 157) | ng/g of lipid | 75.7 | 1.07 | 1.99 | 3.01 | 84.00 |
| 2,3',4,4',5,5'-Hexachlorobiphenyl (PCB 167) | ng/g of lipid | 73.1 | 1.06 | 2.20 | 3.08 | 50.90 |
| Oxychlordane | ng/g of lipid | 66.3 | 13.90 | 24.70 | 34.40 | 159.0 |
| 3,3',4,4',5-Pentachlorobiphenyl (PCB 126) | pg/g of lipid | 66.1 | 18.70 | 32.20 | 48.40 | 721.0 |
| 2,2',3,3',4,4',5,6-Octachlorobiphenyl (PCB 195) | ng/g of lipid | 63.6 | 1.20 | 2.30 | 3.21 | 17.20 |
| 1,2,3,4,6,7,8,9-Octachlorodibenzo-*p*-dioxin (OCDD) | pg/g of lipid | 56.8 | 256.0 | 401.5 | 493.0 | 3280.0 |
| *beta*-Hexachlorocyclohexane | ng/g of lipid | 56.6 | 10.80 | 21.60 | 42.25 | 2850.0 |
| 1,2,3,7,8-Pentachlorodibenzo-*p*-dioxin (PeCDD) | pg/g of lipid | 49.0 | <LD | 7.70 | 10.10 | 36.30 |
| 3,3',4,4',5,5'-Hexachlorobiphenyl (PCB 169) | pg/g of lipid | 43.0 | <LD | 23.20 | 36.15 | 196.0 |

[ Continued next page ]

**S1 Table, continued.**

| **Persistent organic pollutant** | **Units of measurement** | **% ≥LOD** | **median** | **P75** | **P90** | **maximum** |
| --- | --- | --- | --- | --- | --- | --- |
| Dieldrin | ng/g of lipid | 38.5 | <LD | 9.60 | 11.60 | 448.0 |
| 2,2’,4,4’,5-Pentabromodiphenyl ether (BDE 99) | ng/g of lipid | 35.4 | <LD | 6.25 | 11.80 | 692.0 |
| 2,3,4,7,8-Pentachlorodibenzofuran (PeCDF) | pg/g of lipid | 31.9 | <LD | 7.50 | 10.70 | 65.40 |
| Heptachlor epoxide | ng/g of lipid | 30.2 | <LD | 8.10 | 11.10 | 154.0 |
| 2,2',3,5,5',6-Hexachlorobiphenyl (PCB 151) | ng/g of lipid | 29.0 | <LD | 0.40 | 0.70 | 9.30 |
| *p,p'*-Dichlorodiphenyltrichloroethane (DDT) | ng/g of lipid | 26.7 | <LD | 8.40 | 14.40 | 676.0 |
| 1,2,3,4,6,7,8-Heptachlorodibenzofuran (HpCDF) | pg/g of lipid | 26.4 | <LD | 8.70 | 11.50 | 367.0 |
| Perfluorodecanoic acid (PFDeA) | μg/L | 20.9 | <LD | <LD | 0.40 | 3.70 |
| 2,2',4,4',5,6'-Hexabromodiphenyl ether (BDE 154) | ng/g of lipid | 20.0 | <LD | <LD | 1.20 | 40.50 |
| 2,3,3’,4,4’,5,5’-Heptachlorobiphenyl (PCB 189) | ng/g of lipid | 16.8 | <LD | <LD | 0.80 | 12.00 |
| 2,3,7,8-Tetrachlorodibenzo-*p*-dioxin (TCDD) | pg/g of lipid | 14.9 | <LD | <LD | 4.50 | 28.60 |
| 2-(N-Methyl-perfluorooctane sulfonamido) acetic acid (Me-PFOSA-AcOH) | μg/L | 14.9 | <LD | <LD | 0.70 | 62.30 |
| Perfluorooctane sulfonamide (PFOSA) | μg/L | 12.1 | <LD | <LD | 0.20 | 3.10 |
| Mirex | ng/g of lipid | 7.8 | <LD | <LD | <LD | 166.0 |
| 1,2,3,4,7,8-Hexachlorodibenzofuran (HxCDF) | pg/g of lipid | 7.4 | <LD | <LD | <LD | 32.10 |
| Perfluoroundecanoic acid (PFUA) | μg/L | 6.1 | <LD | <LD | <LD | 6.90 |
| 2,2',3,4,4'-Pentabromodiphenyl ether (BDE 85) | ng/g of lipid | 5.4 | <LD | <LD | <LD | 66.90 |
| 2,2',3,3',4,4'-Hexachlorobiphenyl (PCB 128) | ng/g of lipid | 5.4 | <LD | <LD | <LD | 9.20 |
| 1,2,3,6,7,8-Hexachlorodibenzofuran (HxCDF) | pg/g of lipid | 5.3 | <LD | <LD | <LD | 35.90 |
| 2,3',4,4'-Tetrabromodiphenyl ether (BDE 66) | ng/g of lipid | 4.7 | <LD | <LD | <LD | 17.40 |
| 1,2,3,4,7,8-Hexachlorodibenzo-*p*-dioxin (HxCDD) | pg/g of lipid | 2.6 | <LD | <LD | <LD | 7.10 |
| 1,2,3,7,8,9-Hexachlorodibenzo-*p*-dioxin (HxCDD) | pg/g of lipid | 2.5 | <LD | <LD | <LD | 7.60 |
| 3,4,4',5-Tetrachlorobiphenyl (PCB 81) | pg/g of lipid | 2.3 | <LD | <LD | <LD | 7.70 |
| 2-(N-Ethyl-perfluorooctane sulfonamido) acetic acid (Et-PFOSA-AcOH) | μg/L | 2.1 | <LD | <LD | <LD | 4.10 |
| Perfluoroheptanoic acid (PFHpA) | μg/L | 1.9 | <LD | <LD | <LD | 3.80 |
| 2,2',3,4,4',5',6-Heptabromodiphenyl ether (BDE 183) | ng/g of lipid | 1.9 | <LD | <LD | <LD | 21.10 |

[ Continued next page ]

**S1 Table, continued.**

| **Persistent organic pollutant** | **Units of measurement** | **% ≥LOD** | **median** | **P75** | **P90** | **maximum** |
| --- | --- | --- | --- | --- | --- | --- |
| 2,2',4-Tribromodiphenyl ether (BDE 17) | ng/g of lipid | 1.2 | <LD | <LD | <LD | 20.60 |
| 1,2,3,4,6,7,8,9-Octachlorodibenzofuran (OCDF) | pg/g of lipid | 1.0 | <LD | <LD | <LD | 434.0 |
| *o,p'*-DDT | ng/g of lipid | 0.5 | <LD | <LD | <LD | 223.0 |
| Perfluorobutane sulfonic acid (PFBuS) | μg/L | 0.4 | <LD | <LD | <LD | 0.60 |
| 2,3,7,8-Tetrachlorodibenzofuran (TCDF) | pg/g of lipid | 0.3 | <LD | <LD | <LD | 12.30 |
| *gamma*-Hexachlorocyclohexane (Lindane) | ng/g of lipid | 0.2 | <LD | <LD | <LD | 304.0 |
| 1,2,3,7,8-Pentachlorodibenzofuran (PeCDF) | pg/g of lipid | 0.1 | <LD | <LD | <LD | 19.60 |
| 1,2,3,4,7,8,9-Heptachlorodibenzofuran (HpCDF) | pg/g of lipid | 0.1 | <LD | <LD | <LD | 53.70 |
| 1,2,3,7,8,9-Hexachlorodibenzofuran (HxCDF) | pg/g of lipid | 0.0 | <LD | <LD | <LD | 26.90 |
| 2,3,4,6,7,8-Hexachlorodibenzofuran (HxCDF) | pg/g of lipid | 0.0 | <LD | <LD | <LD | 11.60 |
| Perfluorododecanoic acid (PFDoA) | μg/L | 0.0 | <LD | <LD | <LD | 1.00 |
| Aldrin | ng/g of lipid | 0.0 | <LD | <LD | <LD | 58.10 |
| Endrin | ng/g of lipid | 0 | <LD | <LD | <LD | <LD |
