# Supplementary material for: Number of Persistent Organic Pollutants Detected at High Concentrations in Blood Samples of the United States Population
Source: PLoS One. 2016 Aug 10;11(8):e0160432. doi: 10.1371/journal.pone.0160432 (PMC4979965; doi:10.1371/journal.pone.0160432)
Supplement: S2 Table — (DOCX) [file pone.0160432.s003.docx]

**S2 Table. Population characteristics by survey subsample.**

|  | **Total** | | | **PFCs** | | |  | **OCs, PBDEs & PBB 153 compounds** | |  | **PCBs & PCDD/Fs compounds** | |  |
| --- | --- | --- | --- | --- | --- | --- | --- | --- | --- | --- | --- | --- | --- |
|  | (N = 4,739) | | | (N = 1,610) | | |  | (N = 1,585) | |  | (N = 1,544) | |  |
| **Characteristics** | N | (%) |  |  | N | (%) |  | N | (%) |  | N | (%) | ***p-*value** |
| **Gender** |  |  | |  | |  |  |  |  |  |  |  | 0.761 |
| Women | 2467 | (52.1) | | 839 | | (52.1) |  | 835 | (52.7) |  | 793 | (51.4) |  |
| Men | 2272 | (47.9) | | 771 | | (47.9) |  | 750 | (47.3) |  | 751 | (48.6) |  |
| **Age** (years) |  |  | |  | |  |  |  |  |  |  |  |  |
| mean (SD) | 50.4 | (19.5) | | 51.4 | | (19.8) |  | 49.6 | (19.4) |  | 50.3 | (19.4) | 0.036^a^ |
| median | 49.0 |  | | 50.5 | |  |  | 48.0 |  |  | 49.0 |  | 0.041^b^ |
| **Race/ethnicity** |  |  | |  | |  |  |  |  |  |  |  | 0.233 |
| Non-Hispanic white | 2539 | (53.6) | | 860 | | (53.4) |  | 835 | (52.7) |  | 844 | (54.7) |  |
| Mexican American | 951 | (20.1) | | 323 | | (20.1) |  | 325 | (20.5) |  | 303 | (19.6) |  |
| Non-Hispanic black | 948 | (20.0) | | 345 | | (21.4) |  | 316 | (19.9) |  | 287 | (18.6) |  |
| Other Hispanic | 140 | (3.0) | | 38 | | (2.4) |  | 49 | (3.1) |  | 53 | (3.4) |  |
| Other | 161 | (3.4) | | 44 | | (2.7) |  | 60 | (3.8) |  | 57 | (3.7) |  |
| **Educational level** |  |  | |  | |  |  |  |  |  |  |  | 0.361 |
| College or above | 2138 | (45.1) | | 694 | | (43.2) |  | 736 | (46.5) |  | 708 | (45.9) |  |
| High school | 1193 | (25.2) | | 427 | | (26.6) |  | 387 | (24.4) |  | 379 | (24.6) |  |
| < High school | 1399 | (29.5) | | 485 | | (30.2) |  | 460 | (29.1) |  | 454 | (29.5) |  |
| **Poverty income ratio** |  |  | |  | |  |  |  |  |  |  |  | 0.915 |
| >2 | 2394 | (53.6) | | 810 | | (53.1) |  | 797 | (53.9) |  | 787 | (53.7) |  |
| ≤2 | 2075 | (46.4) | | 714 | | (46.9) |  | 682 | (46.1) |  | 679 | (46.3) |  |
| **Body mass index** (kg/m^2^) |  |  | |  | |  |  |  |  |  |  |  |  |
| mean (SD) | 28.4 | (6.3) | | 28.6 | | (6.4) |  | 28.3 | (6.0) |  | 28.4 | (6.4) | 0.433^a^ |
| median | 27.4 |  | | 27.6 | |  |  | 27.4 |  |  | 27.3 |  | 0.593^b^ |
| **Pregnancy^c^** |  |  | |  | |  |  |  |  |  |  |  | 0.886 |
| No | 93 | (3.8) | | 30 | | (3.6) |  | 31 | (3.7) |  | 32 | (4.0) |  |
| Yes | 1768 | (71.7) | | 604 | | (72.0) |  | 597 | (71.5) |  | 567 | (71.5) |  |
| **No. of pregnancies resulting in live births^c^** |  |  | |  | |  |  |  |  |  |  |  |  |
| mean (SD) | 2.8 | (1.9) | | 2.9 | | (2.0) |  | 2.7 | (1.8) |  | 2.8 | (2.0) | 0.157^a^ |
| median | 2.0 |  | | 2.0 | |  |  | 2.0 |  |  | 2.0 |  | 0.263^b^ |
| **Breastfeeding^d,e^** |  |  | |  | |  |  |  |  |  |  |  | 0.323 |
| No | 78 | (4.4) | | 25 | | (4.1) |  | 32 | (5.4) |  | 21 | (3.7) |  |
| Yes | 975 | (55.1) | | 333 | | (55.1) |  | 322 | (53.9) |  | 320 | (56.4) |  |
| **No. of children breastfed^d,e^** |  |  | |  | |  |  |  |  |  |  |  |  |
| mean (SD) | 2.3 | (2.0) | | 2.4 | | (2.0) |  | 2.2 | (1.9) |  | 2.4 | (2.0) | 0.158^a^ |
| median | 2.0 |  | | 2.0 | |  |  | 2.0 |  |  | 2.0 |  | 0.074^b^ |

Unless otherwise specified, *p-*value derived from Pearson's Chi-Square test.

^a^ ANOVA.

^b^ Kruskal–Wallis test.

^c^ Women only.

^d^ Only women with ≥1 pregnancies resulting in live births.

^e^ Breastfed ≥1 month.
