# Supplementary material for: Number of Persistent Organic Pollutants Detected at High Concentrations in Blood Samples of the United States Population
Source: PLoS One. 2016 Aug 10;11(8):e0160432. doi: 10.1371/journal.pone.0160432 (PMC4979965; doi:10.1371/journal.pone.0160432)
Supplement: S3 Table — (DOCX) [file pone.0160432.s004.docx]

**S3 Table. Frequency of subjects with high concentrations of the most detected POPs according to different definitions of ‘high concentration’.**

|  | **Number of the most prevalent POPs at ‘high concentrations’** | | | | | | | | | | | | | | | | |
| --- | --- | --- | --- | --- | --- | --- | --- | --- | --- | --- | --- | --- | --- | --- | --- | --- | --- |
|  |  | **0** | |  | **≥10** | |  | **≥20** | |  | **1 to 5** | |  | **6 to 9** | |  |  |
| **Cutoff point of ‘high concentration’** | **N^a^** | N | (%) |  | N | (%) |  | N | (%) |  | N | (%) |  | N | (%) |  | **GM** |
| **Different cutoff point for each compound** |  |  |  |  |  |  |  |  |  |  |  |  |  |  |  |  |  |
| (No. of participants = 4,739) |  |  |  |  |  |  |  |  |  |  |  |  |  |  |  |  |  |
| POPs detected ≥85% of participants | 37 |  |  |  |  |  |  |  |  |  |  |  |  |  |  |  |  |
| ≥Percentile 90 (top decile) |  | 1,555 | (32.8) |  | 619 | (13.1) |  | 135 | (2.8) |  | 2,190 | (46.2) |  | 375 | (7.9) |  | 3.4 |
| ≥Percentile 80 (top quintile) |  | 559 | (11.8) |  | 1,326 | (28.0) |  | 636 | (13.4) |  | 2,194 | (46.3) |  | 660 | (13.9) |  | 5.3 |
| ≥Percentile 75 (top quartile) |  | 441 | (9.3) |  | 1,776 | (37.5) |  | 852 | (18.0) |  | 1,785 | (37.7) |  | 737 | (15.6) |  | 6.8 |
| POPs detected ≥75% of participants | 44 |  |  |  |  |  |  |  |  |  |  |  |  |  |  |  |  |
| ≥Percentile 90 (top decile) |  | 1,513 | (31.9) |  | 705 | (14.9) |  | 269 | (5.7) |  | 2,113 | (44.6) |  | 408 | (8.6) |  | 3.8 |
| ≥Percentile 80 (top quintile) |  | 538 | (11.4) |  | 1,435 | (30.3) |  | 829 | (17.5) |  | 2,064 | (43.6) |  | 702 | (14.8) |  | 6.0 |
| ≥Percentile 75 (top quartile) |  | 428 | (9.0) |  | 1,925 | (40.6) |  | 1,021 | (21.5) |  | 1,558 | (32.9) |  | 828 | (17.5) |  | 7.8 |
| POPs detected ≥50% of participants | 50 |  |  |  |  |  |  |  |  |  |  |  |  |  |  |  |  |
| ≥Percentile 90 (top decile) |  | 1,446 | (30.5) |  | 766 | (16.2) |  | 350 | (7.4) |  | 2,080 | (43.9) |  | 447 | (9.4) |  | 4.1 |
| ≥Percentile 80 (top quintile) |  | 525 | (11.1) |  | 1,539 | (32.5) |  | 936 | (19.8) |  | 1,899 | (40.1) |  | 776 | (16.4) |  | 6.6 |
| ≥Percentile 75 (top quartile) |  | 409 | (8.6) |  | 2,140 | (45.2) |  | 1,086 | (22.9) |  | 1,400 | (29.5) |  | 790 | (16.7) |  | 8.7 |
| **Participants ≥percentile 75 of [TEQ]** |  |  |  |  |  |  |  |  |  |  |  |  |  |  |  |  |  |
| **and different cutoff point for compounds** |  |  |  |  |  |  |  |  |  |  |  |  |  |  |  |  |  |
| (No. of participants = 1,183) |  |  |  |  |  |  |  |  |  |  |  |  |  |  |  |  |  |
| POPs detected ≥85% of participants | 33 |  |  |  |  |  |  |  |  |  |  |  |  |  |  |  |  |
| ≥Percentile 90 (top decile) |  | 110 | (9.3) |  | 356 | (30.1) |  | 80 | (6.8) |  | 568 | (48.0) |  | 149 | (12.6) |  | 5.0 |
| ≥Percentile 80 (top quintile) |  | 6 | (0.5) |  | 832 | (70.3) |  | 248 | (21.0) |  | 245 | (20.7) |  | 100 | (8.5) |  | 10.7 |
| ≥Percentile 75 (top quartile) |  | 1 | (0.1) |  | 897 | (75.8) |  | 426 | (36.0) |  | 205 | (17.3) |  | 80 | (6.8) |  | 12.4 |
| POPs detected ≥75% of participants | 38 |  |  |  |  |  |  |  |  |  |  |  |  |  |  |  |  |
| ≥Percentile 90 (top decile) |  | 106 | (9.0) |  | 428 | (36.2) |  | 136 | (11.5) |  | 526 | (44.5) |  | 123 | (10.4) |  | 6.1 |
| ≥Percentile 80 (top quintile) |  | 6 | (0.5) |  | 869 | (73.5) |  | 548 | (46.3) |  | 199 | (16.8) |  | 109 | (9.2) |  | 12.5 |
| ≥Percentile 75 (top quartile) |  | 1 | (0.1) |  | 937 | (79.2) |  | 715 | (60.4) |  | 154 | (13.0) |  | 91 | (7.7) |  | 15.3 |
| POPs detected ≥50% of participants | 41 |  |  |  |  |  |  |  |  |  |  |  |  |  |  |  |  |
| ≥Percentile 90 (top decile) |  | 103 | (8.7) |  | 446 | (37.7) |  | 165 | (13.9) |  | 483 | (40.8) |  | 151 | (12.8) |  | 6.7 |
| ≥Percentile 80 (top quintile) |  | 6 | (0.5) |  | 885 | (74.8) |  | 628 | (53.1) |  | 187 | (15.8) |  | 105 | (8.9) |  | 13.7 |
| ≥Percentile 75 (top quartile) |  | 1 | (0.1) |  | 955 | (80.7) |  | 757 | (64.0) |  | 137 | (11.6) |  | 90 | (7.6) |  | 16.8 |

GM: geometric mean. Geometric means are calculated for subjects with ≥1 of respective most prevalent POPs at ‘high concentrations’ according to each cutoff point for ‘high concentration’.

^a^ Number of POPs included in the analyses.
