# Supplementary material for: Number of Persistent Organic Pollutants Detected at High Concentrations in Blood Samples of the United States Population
Source: PLoS One. 2016 Aug 10;11(8):e0160432. doi: 10.1371/journal.pone.0160432 (PMC4979965; doi:10.1371/journal.pone.0160432)
Supplement: S4 Table — S4ATable. Associations between sociodemographic characteristics and having one or more POPs with concentrations in the upper decile. S4B Table. Associations between sociodemographic characteristics and having one or more OCs, PBDEs and PBB 153 with concentrations in the upper decile. (DOCX) [file pone.0160432.s005.docx]

**S4A Table. Associations between sociodemographic characteristics**

**and having one or more POPs with concentrations in the upper decile.**

| **Characteristics** | **Model 1** | | | |  | **Model 2** | | | |  |
| --- | --- | --- | --- | --- | --- | --- | --- | --- | --- | --- |
|  | **OR** | **(95% CI)** | | ***p-*value** |  | **OR** | **(95% CI)** | | ***p-*value** |  |
| **Gender** |  |  |  |  |  |  |  |  |  |  |
| Women | 1.00 |  |  |  |  | 1.00 |  |  |  |  |
| Men | 1.87 | (1.64, | 2.13) | <0.001 |  | 1.88 | (1.64, | 2.14) | <0.001 |  |
| **Age** (years) | 1.04 | (1.04, | 1.04) | <0.001 |  | 1.04 | (1.04, | 1.04) | <0.001 |  |
| **Race/ethnicity** |  |  |  |  |  |  |  |  |  |  |
| Non-Hispanic White | 1.00 |  |  | <0.001 |  | -- |  |  |  |  |
| Mexican American | 0.74 | (0.63, | 0.88) |  |  |  |  |  |  |  |
| Non-Hispanic Black | 1.10 | (0.93, | 1.31) |  |  |  |  |  |  |  |
| Other Hispanic | 0.73 | (0.50, | 1.05) |  |  |  |  |  |  |  |
| Other | 1.32 | (0.90, | 1.91) |  |  |  |  |  |  |  |
| **Educational level** |  |  |  |  |  |  |  |  |  |  |
| College or Above | 1.00 |  |  | 0.616 |  | 1.00 |  |  | 0.460 |  |
| High School | 0.93 | (0.79, | 1.09) |  |  | 0.95 | (0.81, | 1.12) |  |  |
| < High School | 0.95 | (0.81, | 1.11) |  |  | 1.07 | (0.90, | 1.28) |  |  |
| **Poverty income ratio** |  |  |  |  |  |  |  |  |  |  |
| >2 | 1.00 |  |  |  |  | 1.00 |  |  |  |  |
| ≤2 | 1.11 | (0.97, | 1.27) | 0.137 |  | 1.17 | (1.02, | 1.35) | 0.026 |  |
| **Body mass index** (kg/m^2^) |  |  |  |  |  |  |  |  |  |  |
| Normal weight | 1.00 |  |  | <0.001 | ^a^ | 1.00 |  |  | <0.001 | ^a^ |
| Overweight | 0.81 | (0.68, | 0.95) |  |  | 0.83 | (0.70, | 0.98) |  |  |
| Obesity | 0.71 | (0.61, | 0.84) |  |  | 0.73 | (0.62, | 0.86) |  |  |
| **Pregnancy^b^** |  |  |  |  |  |  |  |  |  |  |
| No | 1.00 |  |  |  |  | 1.00 |  |  |  |  |
| Yes | 0.77 | (0.48, | 1.23) | 0.275 |  | 0.73 | (0.45, | 1.17) | 0.189 |  |
| **No. pregnancies resulting in live births^b^** | 1.04 | (0.98, | 1.11) | 0.224 |  | 1.00 | (0.93, | 1.07) | 0.888 |  |
| **Breastfeeding^c,d^** |  |  |  |  |  |  |  |  |  |  |
| No | 1.00 |  |  |  |  | 1.00 |  |  |  |  |
| Yes | 0.64 | (0.37, | 1.11) | 0.113 |  | 0.62 | (0.36, | 1.07) | 0.087 |  |
| **No. children breastfed^c,d^** | 0.97 | (0.85, | 1.10) | 0.597 |  | 0.96 | (0.85, | 1.10) | 0.585 |  |

Model 1: adjusted by age, gender and body mass index.

Model 2: adjusted by age, gender, body mass index and race/ethnicity.

Number of POPs at high concentrations: number of POPs whose serum concentrations were ≥percentile 90.

Unless otherwise specified, *p*-value derived from Wald’s test.

^a^ Multivariate analogue of Mantel’s extension test for linear trend.

^b^ Women only.

^c^ Only among women with ≥1 pregnancies resulting in live births and, in the two models, further adjusted by such number of pregnancies.

^d^ Breastfed ≥1 month.

**S4B Table. Associations between sociodemographic characteristics**

**and having one or more OCs, PBDEs and PBB 153 with concentrations in the upper decile.**

| **Characteristics** | **Model 1** | | | |  | **Model 2** | | | |  |
| --- | --- | --- | --- | --- | --- | --- | --- | --- | --- | --- |
|  | **OR** | **(95% CI)** | | ***p-*value** |  | **OR** | **(95% CI)** | | ***p-*value** |  |
| **Gender** |  |  |  |  |  |  |  |  |  |  |
| Women | 1.00 |  |  |  |  | 1.00 |  |  |  |  |
| Men | 1.17 | (1.04, | 1.33) | 0.013 |  | 1.17 | (1.03, | 1.33) | 0.015 |  |
| **Age** (years) | 1.02 | (1.02, | 1.03) | <0.001 |  | 1.02 | (1.02, | 1.03) | <0.001 |  |
| **Race/ethnicity** |  |  |  |  |  |  |  |  |  |  |
| Non-Hispanic White | 1.00 |  |  | <0.001 |  | -- |  |  |  |  |
| Mexican American | 1.41 | (1.20, | 1.66) |  |  |  |  |  |  |  |
| Non-Hispanic Black | 0.76 | (0.63, | 0.90) |  |  |  |  |  |  |  |
| Other Hispanic | 0.85 | (0.57, | 1.27) |  |  |  |  |  |  |  |
| Other | 0.60 | (0.41, | 0.89) |  |  |  |  |  |  |  |
| **Educational level** |  |  |  |  |  |  |  |  |  |  |
| College or Above | 1.00 |  |  | 0.002 | ^a^ | 1.00 |  |  | 0.069 |  |
| High School | 1.01 | (0.86, | 1.19) |  |  | 0.99 | (0.84, | 1.16) |  |  |
| < High School | 1.28 | (1.11, | 1.49) |  |  | 1.19 | (1.01, | 1.40) |  |  |
| **Poverty income ratio** |  |  |  |  |  |  |  |  |  |  |
| >2 | 1.00 |  |  |  |  | 1.00 |  |  |  |  |
| ≤2 | 1.19 | (1.04, | 1.35) | 0.011 |  | 1.15 | (1.01, | 1.32) | 0.038 |  |
| **Body mass index** (kg/m^2^) |  |  |  |  |  |  |  |  |  |  |
| Normal weight | 1.00 |  |  | 0.013 | ^a^ | 1.00 |  |  | 0.006 | ^a^ |
| Overweight | 0.96 | (0.83, | 1.13) |  |  | 0.93 | (0.80, | 1.09) |  |  |
| Obesity | 0.82 | (0.70, | 0.96) |  |  | 0.80 | (0.68, | 0.94) |  |  |
| **Pregnancy^b^** |  |  |  |  |  |  |  |  |  |  |
| No | 1.00 |  |  |  |  | 1.00 |  |  |  |  |
| Yes | 1.34 | (0.76, | 2.14) | 0.279 |  | 1.17 | (0.67, | 2.02) | 0.588 |  |
| **No. pregnancies resulting in live births^b^** | 1.13 | (1.07, | 1.20) | <0.001 |  | 1.04 | (0.98, | 1.11) | 0.168 |  |
| **Breastfeeding^c,d^** |  |  |  |  |  |  |  |  |  |  |
| No | 1.00 |  |  |  |  | 1.00 |  |  |  |  |
| Yes | 0.86 | (0.51, | 1.46) | 0.571 |  | 0.71 | (0.41, | 1.22) | 0.213 |  |
| **No. children breastfed^c,d^** | 1.02 | (0.91, | 1.13) | 0.773 |  | 0.96 | (0.86, | 1.07) | 0.438 |  |

Model 1: adjusted by age, gender and body mass index.

Model 2: adjusted by age, gender, body mass index and race/ethnicity.

Unless otherwise specified, *p-*value derived from Wald’s test.

^a^ Multivariate analogue of Mantel’s extension test for linear trend.

^b^ Women only.

^c^ Only among women with ≥1 pregnancies resulting in live births and, in the two models, further adjusted by such number of pregnancies.

^d^ Breastfed ≥1 month.
