# Supplementary material for: Number of Persistent Organic Pollutants Detected at High Concentrations in Blood Samples of the United States Population
Source: PLoS One. 2016 Aug 10;11(8):e0160432. doi: 10.1371/journal.pone.0160432 (PMC4979965; doi:10.1371/journal.pone.0160432)
Supplement: S5 Table — S5A Table. Serum pooled concentrations of POPs most detected for the three most recent NHANES Surveys periods analyzed. S5B Table. Serum concentrations of four perfluorinated compounds (μg/L) most detected for the most recent NHANES Surveys periods analyzed. (DOCX) [file pone.0160432.s006.docx]

**S5A Table. Serum pooled concentrations^a^ of POPs most detected for the three most recent NHANES Surveys periods analyzed.**

|  | **2003-2004^b^** | | **2005-2006** | | **2007-2008** | |
| --- | --- | --- | --- | --- | --- | --- |
| **Persistent organic pollutant** | **mean** | **(SD)** | **mean** | **(SD)** | **mean** | **(SD)** |
| 2,2',3,4,4',5' and 2,3,3',4,4',6-Hexachlorobiphenyl (PCB 138 & 158) | 29.38 | (24.6) | 14.60 | (17.5) | 23.26 | (22.1) |
| 2,3',4,4',5-Pentachlorobiphenyl (PCB 118) | 11.48 | (10.4) | 7.48 | (9.6) | 8.99 | (9.6) |
| 2,2',4,4',5,5'-Hexachlorobiphenyl (PCB 153) | 39.47 | (32.9) | 25.11 | (29.8) | 30.69 | (28.8) |
| 2,2',3,4,4',5,5'-Heptachlorobiphenyl (PCB 180) | 32.93 | (26.9) | 20.49 | (24.7) | 25.11 | (26.9) |
| 2,2',3,5'-Tetrachlorobiphenyl (PCB 44) | 2.26 | (0.7) | 0.20 | (0.1) | 0.12 | (0.1) |
| 2,4,4',5-Tetrachlorobiphenyl (PCB 74) | 8.73 | (7.7) | 5.83 | (7.6) | 6.01 | (5.8) |
| *p,p'*-Dichlorodiphenyldichloroethene (DDE) | 731.3 | (766) | 702.6 | (1212) | 585.6 | (909) |
| 2,2',4,4',5-Pentachlorobiphenyl (PCB 99) | 6.83 | (5.6) | 4.21 | (4.6) | 4.76 | (4.4) |
| 2,2',3,3',4,4',5-Heptachlorobiphenyl (PCB 170) | 11.49 | (9.4) | 7.38 | (8.9) | 9.93 | (9.2) |
| 2,2',3,4',5,5',6-Heptachlorobiphenyl (PCB 187) | 9.66 | (8.2) | 6.47 | (8.4) | 8.44 | (8.8) |
| 2,4,4'-Trichlorobiphenyl (PCB 28) | 5.48 | (1.8) | 1.95 | (2.3) | 1.36 | (1.7) |
| 2,2',4,5'-Tetrachlorobiphenyl (PCB 49) | 1.46 | (0.5) | 0.15 | (0.1) | 0.11 | (0.0) |
| 2,2',3,4',5,5'-Hexachlorobiphenyl (PCB 146) | 4.77 | (4.3) | 3.18 | (4.3) | 4.02 | (4.2) |
| Hexachlorobenzene | 16.76 | (4.3) | 8.78 | (4.1) | 11.23 | (4.7) |
| 2,2',5,5'-Tetrachlorobiphenyl (PCB 52) | 3.05 | (1.0) | 0.27 | (0.2) | 0.17 | (0.2) |
| 2,2',3,3',4,4',5,6' and 2,2',3,4,4',5,5',6-Octachlorobiphenyl (PCB 196 & 203) | 6.01 | (4.8) | 5.02 | (6.1) | 6.31 | (6.1) |
| 2,3,3',4,4'-Pentachlorobiphenyl (PCB 105) | 2.24 | (2.1) | 1.41 | (1.8) | 1.85 | (2.1) |
| 2,2',4,4'-Tetrabromodiphenyl ether (BDE 47) | 34.46 | (30.2) | 47.05 | (41.2) | 34.19 | (25.1) |
| 2,3,3',4,4',5-Hexachlorobiphenyl (PCB 156) | 6.06 | (6.5) | 3.45 | (4.2) | 4.90 | (4.8) |
| 2,2',3,3',4,5,5',6-Octachlorobiphenyl (PCB 199) | 7.71 | (6.8) | 5.72 | (7.6) | 7.14 | (7.4) |
| 2,2',3,4,4',5',6-Heptachlorobiphenyl (PCB 183) | 3.11 | (2.4) | 1.97 | (2.2) | 2.59 | (2.5) |
| 2,2',4,5,5'-Pentachlorobiphenyl (PCB 101) | 2.05 | (0.8) | 0.38 | (0.3) | 0.29 | (0.5) |
| 2,2',3,3',4,4',5,5'-Octachlorobiphenyl (PCB 194) | 7.33 | (5.9) | 4.67 | (5.9) | 6.29 | (6.1) |
| 2,3',4,4'-Tetrachlorobiphenyl (PCB 66) | 1.78 | (1.1) | 0.86 | (1.0) | 0.87 | (0.8) |
| 2,2',3,3',4,5',6'-Heptachlorobiphenyl (PCB 177) | 2.89 | (2.5) | 1.29 | (1.6) | 1.67 | (1.7) |
| 1,2,3,4,6,7,8-Heptachlorodibenzo-*p*-dioxin (HpCDD) | 36.71 | (17.6) | 28.11 | (16.7) | 25.46 | (14.5) |
| 2,2',4,4',6-Pentabromodiphenyl ether (BDE 100) | 6.80 | (6.4) | 9.29 | (8.7) | 7.14 | (5.5) |
| 2,2',3,4,5'-Pentachlorobiphenyl (PCB 87) | 1.04 | (0.4) | 0.21 | (0.2) | 0.19 | (0.3) |
| 2,2',3,4',5',6-Hexachlorobiphenyl (PCB 149) | 0.75 | (0.3) | 0.18 | (0.1) | 0.15 | (0.2) |
| 2,2',4,4',5,5'-Hexabromodiphenyl ether (BDE 153) | 10.06 | (11.3) | 11.63 | (9.1) | 12.06 | (9.2) |
| 2,2',4,4',5,5'-Hexabromobiphenyl (BB 153) | 4.60 | (4.7) | 5.05 | (11.0) | 5.01 | (9.9) |
| 2,3,3',4',6-Pentachlorobiphenyl (PCB 110) | 1.48 | (0.6) | 0.19 | (0.1) | 0.16 | (0.3) |
| 2,2',3,3',4,4',5,5',6-Nonachlorobiphenyl (PCB 206) | 5.08 | (4.6) | 3.58 | (5.4) | 4.04 | (4.4) |

Values for participants ≥20 years old.

SD: standard deviation of arithmetic mean.

^a^ All compounds measured in ng/g of lipid, except HpCDD (in pg/g of lipid). ^b^ Values for all 4,739 participants.

**S5B Table. Serum concentrations of four perfluorinated compounds (μg/L) most detected for the most recent NHANES Surveys periods analyzed.**

|  | **2003-2004^a^** | | **2005-2006** | | **2007-2008** | | **2009-2010** | | **2011-2012** | |
| --- | --- | --- | --- | --- | --- | --- | --- | --- | --- | --- |
| **Persistent organic pollutant** | **GM** | **(SD)** | **GM** | **(SD)** | **GM** | **(SD)** | **GM** | **(SD)** | **GM** | **(SD)** |
| Perfluorooctane sulfonic acid (PFOS) | 20.11 | (1.7) | 16.68 | (2.2) | 13.79 | (2.3) | 9.26 | (2.3) | 6.86 | (2.5) |
| Perfluorooctanoic acid (PFOA) | 3.64 | (1.7) | 3.53 | (2.2) | 4.03 | (1.9) | 2.89 | (1.9) | 2.04 | (2.0) |
| Perfluorononanoic acid (PFNA) | 0.94 | (1.8) | 1.06 | (2.1) | 1.22 | (1.9) | 1.25 | (1.9) | 0.94 | (2.0) |
| Perfluorohexane sulfonic acid (PFHxS) | 1.75 | (1.9) | 1.47 | (3.0) | 1.90 | (2.6) | 1.49 | (2.5) | 1.20 | (2.6) |

Values for participants ≥20 years old.

GM: geometric mean.

SD: standard deviation of geometric mean.

^a^ Values for all 4,739 participants.
